# Lung Cancer Characteristics in the World Trade Center Environmental Health Center

**DOI:** 10.3390/ijerph18052689

**Published:** 2021-03-07

**Authors:** Nedim Durmus, Sultan Pehlivan, Yian Zhang, Yongzhao Shao, Alan A. Arslan, Rachel Corona, Ian Henderson, Daniel H. Sterman, Joan Reibman

**Affiliations:** 1 Department of Medicine, Division of Pulmonary Medicine, School of Medicine (SOM), New York University, New York, NY 10016, USA; Nedim.Durmus@nyulangone.org (N.D.); sultan.pehlivan@nyulangone.org (S.P.); ian.henderson@nyulangone.org (I.H.); daniel.sterman@nyulangone.org (D.H.S.); 2 World Trade Center Environmental Health Center, NYC H+HC, New York, NY 10016, USA; yian.zhang@nyulangone.org (Y.Z.); yongzhao.shao@nyulangone.org (Y.S.); alan.arslan@nyulangone.org (A.A.A.); rachel.corona@downstate.edu (R.C.); 3 Department of Population Health, Division of Biostatistics, School of Medicine (SOM), New York University, New York, NY 10016, USA; 4 Department of Environmental Medicine, School of Medicine (SOM), New York University, New York, NY 10016, USA; 5 NYU Perlmutter Comprehensive Cancer Center, New York, NY 10016, USA; 6 Department of Obstetrics and Gynecology, School of Medicine (SOM), New York University, New York, NY 10016, USA

**Keywords:** WTC Environmental Health Center, World Trade Center, September 11th, lung cancer, cancer characteristics

## Abstract

The destruction of the World Trade Center (WTC) towers on 11 September 2001 resulted in acute and chronic dust and fume exposures to community members, including local workers and residents, with well-described aerodigestive adverse health effects. This study aimed to characterize lung cancer in the WTC Environmental Health Center (WTC EHC) focusing on gender and smoking history. WTC EHC patients undergo an initial evaluation that includes WTC exposure information, demographics, and tobacco use. Detailed cancer characteristics are recorded from pathology reports. As of 31 December 2019, 248 WTC EHC patients had a diagnosis of lung cancer. More patients with lung cancer were women (57%) compared to men (43%). Many cases (47% women, 51% men) reported acute dust cloud exposure. Thirty-seven percent of lung cancer cases with available smoking history were never-smokers (≤1 pack-years) and 42% had a ≤5 pack-year history. The median age of cancer diagnosis in never-smoking women was 61 years compared to 66 years in men. Adenocarcinoma was more common in never-smokers compared to ever-smokers (72% vs. 65%) and in women compared to men (70% vs. 65%). We provide an initial description of lung cancers in local community members with documented exposure to the WTC dust and fumes.

## 1. Introduction

The destruction of the World Trade Center (WTC) towers on 11 September 2001 exposed local workers, residents, and those passing by (community members), as well as those involved in rescue and recovery, to potentially extensive inhalation of the WTC dust and fumes [1,2,3]. The WTC Environmental Health Center (WTC EHC) was created in response to local community requests in the years after 11 September. With the advent of the James Zadroga 11 September Health and Compensation Act of 2010 (H.R. 847), the WTC EHC was included as the Center of Excellence for community members (defined as “Survivors”) [4]. Patients self-refer into this program and under law, inclusion requires defined WTC dust/fume exposure and the presence of a “Certifiable condition” [2,4]. Virtually all cancers were added as “Certifiable conditions” in 2012 [5].

We, as well as other researchers, demonstrated that WTC acute and chronic exposures are significantly associated with many adverse health conditions in adults and children, particularly aerodigestive disorders [2,6,7,8,9,10,11,12,13,14,15]. Increased cancer rates (e.g., thyroid cancer, prostate cancer) and decreased rates for lung cancer have been described in the WTC Responders and WTC Survivors [16,17,18,19,20], with numerous cancers included in the list of WTC-related diseases in 2012 [5]. Numerous known carcinogens have been described in the WTC dust and fumes. These have been well-summarized in review papers and include asbestos, silica, heavy metals, as well as polycyclic aromatic hydrocarbons, polychlorinated biphenyls, polychlorinated dibenzofurans, and dioxins. In addition, benzene, and other volatile organic compounds were reported from jet fuels [3,21,22,23,24,25,26]. Because of the inhalation of dust and fumes containing these carcinogens, as well as the identification of persistent particles thought to be derived from WTC dust in the lungs of responders as well as community members [27,28], the risk of lung cancer in exposed populations has been a major concern. Animal studies have suggested potential for carcinogenicity since WTC dust exposure in mice is associated with inflammation, oxidative stress, and epigenetic changes in the lung [29]. Although lung cancer is a leading cause of cancer deaths in the general population among men as well as women [30,31,32,33], the WTC literature on lung cancer in women is limited particularly because WTC responders are mostly men. As such, there is an important knowledge gap about cancers, including lung cancer in women with WTC dust/fume exposure.

Previously, we described overall cancer characteristics in the WTC EHC [34]. Lung cancer is the third most frequent cancer in our community population (9%) and the second most frequent cancer in women (11%). In contrast to responder studies, about half of WTC EHC enrollees are women. In addition to information on WTC exposure information, the WTC EHC database has detailed data on tobacco smoking, a well-known risk factor for lung cancer.

The objective of this paper is to provide descriptive lung cancer characteristics among the WTC “Survivors,” including an examination of potential differences by gender and history of tobacco use. We report a case series of the lung cancers identified in the WTC EHC as of 31 December 2019 and the characteristics of these cancers. To our knowledge, this is the first study reporting detailed lung cancer characteristics in the WTC-exposed population of local community members.

## 2. Methods

### 2.1. Clinical and Lung Cancer Characteristics in Patients in the WTC EHC

Patients self-refer into the WTC EHC under rules now defined by the federal government [4,35]. These rules include specific geographic boundaries (roughly south of Houston Street and some western areas of Brooklyn) and time periods (from 11 September 2001 to 31 July 2002) during which a community member can be considered exposed. In addition, the member must have a “certifiable” condition. Members are included in the WTC EHC clinical database which includes basic demographic information, WTC dust and other exposures collected using detailed questionnaires, and the clinical characteristics of the WTC-certifiable conditions [4]. In this study, we classified exposure to the WTC dust on 11 September 2001 as dust cloud exposure and also classified exposure groups as clean-up workers, residents, students, local workers, and others. Detailed data on WTC exposure and other important exposures, such as smoking history, are also captured, allowing for analyses of the impact of smoking and other factors. Tobacco use was defined as never (≤1 pack-year; p-y), former (>1 p-y, stopped) and current (>1 p-y, continue) at enrollment. However, there were too few current smokers among lung cancer patients (*n* = 14) to make a statistical comparison, and thus we merged the groups of former (*n* = 113) and current (*n* = 14) smokers and categorized tobacco use among lung cancer patients as never-smokers, those reporting a ≤1 p-y (*n* = 76) and ever-smokers, those reporting >1 p-y (*n* = 127). Tobacco use information was not available for lung cancer cases from one clinical site of the WTC EHC (*n* = 45).

### 2.2. Lung Cancer Characteristics

The cancer characteristics of lung cancer patients are derived from the newly developed WTC EHC Pan-Cancer Database (PCDB), which was created to capture information on all cancer types in the WTC EHC and to interface with the current WTC EHC clinical databases [36]. We use REDCap as a secure Federal Information Security Modernization Act and Health Insurance Portability and Accountability Act -compliant environment to support data capture for the WTC EHC PCDB [37,38]. The three main domains of the database include patient demographics, cancer characteristics, and cancer biomarker information [36].

We obtained information on lung cancer characteristics from the WTC EHC PCDB, which includes data extracted from pathology reports and available medical records. All available information are carefully reviewed by a pathologist and clinicians at the WTC EHC. Lung cancer characteristics such as age at diagnosis, anatomic location of tumor (ICD-10 classification), tumor size, tumor grade, tumor histology (ICD-O-3 code), and TNM (Tumor, Node, Metastases) stage are recorded for each cancer case. Second primary tumors, defined according the International Association of Cancer Registries and International Agency for Research on Cancer (IACR/IARC) criteria are also recorded [39]. Importantly, the location of the medical facility where the biopsies and surgeries were performed is included in the database in order to enable future tumor sample procurement.

Subjects were asked to provide their informed consent for inclusion before they participated in the study. The study was conducted in accordance with the Declaration of Helsinki, and the protocol was approved by the New York University School of Medicine Institutional Review Board (IRB number: i06-1). Patients with lung cancer were analyzed after removal of personal identifiers with IRB approval to review de-identified data (IRB number: i06-1_MOD49). Documentation of consent to be re-contacted is included for subsequent studies.

### 2.3. Data Analysis

Descriptive statistics were used to summarize WTC exposure and demographic characteristics of lung cancer patients including median and range for continuous variables and counts and percentages for binary or categorical variables. Tumor characteristics were compared by sex and smoking history using chi-square tests and Fisher’s exact test for categorical variables and Mann–Whitney test for continuous variables. Distribution of age of diagnosis for every 5-year period stratified by sex and smoking history was summarized using bar graphs. Statistical software *R*-3.6.3 was utilized to conduct statistical analyses.

## 3. Results

### 3.1. Participants

We identified 11,038 patients who were enrolled in the WTC EHC between May 2002 and 31 December 2019. Among them, 2840 patients had any type of cancer including 248 with a primary lung cancer diagnosis. Fifteen patients had more than one primary lung cancer diagnosis. Lung cancer was the second most frequent cancer (after breast cancer) in women accounting for 11% of female cancers, whereas it was the third most frequent cancer (after prostate cancer and lymphomas) in men, accounting for 7% of all male cancers patients in the WTC EHC population [34].

### 3.2. WTC Exposure and Demographic Characteristic in Lung Cancer Patients in the WTC EHC Stratified by Smoking History

Tobacco smoking is a major risk factor for lung cancers. We summarize basic exposure and demographic characteristics of lung cancer patients categorized by smoking status in Table 1. Of the 248 patients with lung cancer in the WTC EHC, 203 patients had available smoking history, 119 were women (59%), and 84 (41%) were men. Thirty-seven percent (*n* = 76) were never-smokers (≤1 p-y) whereas 63% (*n* = 127) were ever-smokers (>1 p-y). The median age of lung cancer diagnosis was 62 for never-smokers (range 38–88) and 65 years for ever-smokers (range 34–89). Ever smokers had diverse race/ethnicity composition with 56% White, 23% Asian, and 14% Black or African American. Distribution of race/ethnicity varied by smoking status with a high proportion of Asians (46%) who were never-smokers and many White (66%) who were ever-smokers. Many patients had a low-income (56%) and 38% had an educational status of high school or less. Nearly half of the patients (49%) were exposed to the dust cloud on 11 September 2001 (Table 1).

### 3.3. Lung Cancer Characteristics by Smoking History in the WTC EHC

Overall lung cancer characteristics stratified by smoking history for the first primary lung tumor are shown in Table 2. The majority of the lung cancers (90%) had a histologic type consistent with non-small cell carcinoma, (adenocarcinoma (71%), squamous cell carcinoma (10%), unidentified subtype of non-small cell carcinoma (6%), and large cell carcinoma (1%)). Only 2% of the cancers were small cell carcinoma and 6% were carcinoid tumors. Among all histologic types of lung cancers, 17% were well-differentiated (Grade 1), 21% were moderately-differentiated (grade 2) and 21% were poorly-differentiated (Grade 3). Although the primary cancers were distributed throughout the lung, the right upper lobe (25%) was the most common location of primary lung cancer. Most patients had only one lesion (90%), however 8% had two primary lesions, and 2% had three or more lesions. (Table 2).

The median tumor size at diagnosis was 1.8 cm (range 0.2–11.5 cm) and 42% of patients had less than 3 cm in greatest dimension (T1). No regional lymph node metastases were identified in 55% of the patients. Evidence of regional lymph node involvement was observed in 25% of lung cancer patients. Fifteen percent of the patients had distant metastasis at diagnosis. Using the TNM staging system, at the time of diagnosis, 42% of patients were Stage I, 11% were Stage II, 12% were Stage III, and 15% were Stage IV (Table 4).

There was no significant difference in cancer characteristics stratified by smoking history except histological type. Never-smokers had a higher proportion of adenocarcinoma compared to ever-smokers (79% and 65%, respectively) and carcinoid tumors (9% and 5%, respectively). As expected, ever-smokers had a higher proportion of squamous cell carcinoma compared to never-smokers (16% and 5%, respectively) (*p* = 0.017) (Table 2).

### 3.4. Gender and Lung Cancer in the WTC EHC

The large number of women in the WTC EHC overall and among the lung cancer patients (57%) provided an opportunity to describe lung cancer characteristics in women and to determine potential gender differences of lung cancer characteristics among the WTC survivors. We compared characteristics of lung cancer in women vs. men (Table 3). Women in the WTC EHC with lung cancer were slightly younger on 11 September 2001 compared to men with lung cancer (median, 49.2 vs. 52.7 years respectively; *p* = 0.002) with a higher proportion of women under age of 40 years on that date compared with men (52% vs. 38% respectively; *p* = 0.02). The median age of lung cancer diagnosis was 62 years for women (range 34–85) and 66 years for men (range 38–89; *p* = 0.002). More women lung cancer patients were Black or African-American compared to men (18% vs. 7%) and fewer women compared to men were Hispanic (4% vs. 12%). There were no statistically significant differences in BMI, income, or education between female and male lung cancer patients. Many female lung cancer patients were local residents (42%), whereas most male lung cancer patients were local workers (67%) (Table 2). More women (41%) were never-smokers compared to men (17%, *p* < 0.001) and more men (54%) had a ≥ 5 p-y smoking history compared to women (26%, (*p* < 0.001) (Table 3).

### 3.5. Lung Cancer Characteristics by Gender in the WTC EHC

A number of differences in lung cancer characteristics were noted between women and men (Table 4). Differences were noted in primary tumor location, with 22% of women and 28% of men presenting with cancer in the right upper lobe (*p* = 0.01). Differences were also noted in the primary tumor size classification with more women compared to men presenting with T1 lesions (46% and 37%, respectively) (*p* = 0.006) and a smaller median tumor size at diagnosis in women (1.7 cm) compared to men (2 cm) (*p* = 0.03). There were no significant differences by lymph node involvement, distant metastasis, and stage at diagnosis. Adenocarcinoma was the main histologic type in both female and male patients (75% and 65%, respectively). However, carcinoid tumors were more frequent in women compared to men (9% and 3%, respectively), and squamous cell carcinoma was less common in women compared to men (6% and 17%, respectively, *p* = 0.001) (Table 4).

**Figure 1:**
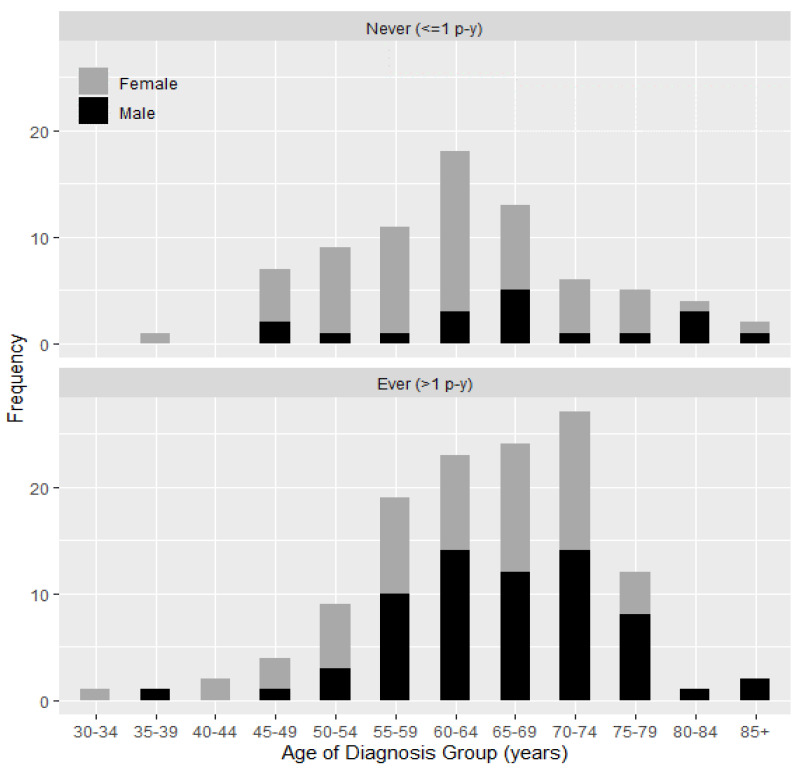
Distribution of age of lung cancer diagnosis in women and men by smoking history.

Figure 1 presents the distribution of age at lung cancer diagnosis for men and women by smoking history. More than three quarters of lung cancers developed among never-smokers were in women (76%; *p* < 0.001). Among never-smokers, there was a higher proportion of women than men at earlier ages of diagnosis (median age at diagnosis, 61 and 66 years, respectively). In contrast, among ever-smokers, there was a higher proportion of men than women at later ages of diagnosis (median age at diagnosis, 67 and 64 years, respectively) (Figure 1).

Distribution of age of lung cancer diagnosis in never-smokers and ever-smokers in women and men are also shown in Supplemental Figure S1 (Figure S1).

### 3.6. Multiple Primary Cancers

Sixty-four lung cancer patients had a second primary tumor. The most common second primary cancer sites included lung (23%), thyroid (19%), prostate (16%), and breast (12%) (Table S1). Among patients with two primary lung cancers, more tumors were diagnosed in the right (54%) compared to the left lung (43%), with two bilateral lung cancer cases. Second primary lung cancer most common characteristics were moderately differentiated (27%), TNM stage I tumors with histology of adenocarcinoma (60%) (Table S2).

## 4. Discussion

The destruction of the WTC towers on 11 September 2001 exposed local residents, workers, and individuals in the area to dust and fumes that included known and suspected carcinogens. Lung cancer is the leading cause of cancer death [40] in the general population, however, the characteristics of lung cancers diagnosed in local community members have not been previously described. In this first descriptive study focusing on lung cancer, we describe the characteristics of lung cancer in both women and men enrolled in the WTC EHC program.

The risk of lung cancer in those exposed to the WTC environmental disaster remains incompletely understood. In an eight-year follow-up study in the firefighters exposed to WTC dust, the lung cancer rate was lower than expected when compared to a pooled cohort of firefighters from San Francisco, Chicago, and Philadelphia [41]. Studies in responders found that the standardized incidence rate of lung cancer was similar to the general population [16]. Little data about the risk of lung cancer is available for WTC-exposed community members. In a 10-year follow-up study performed by the NYC Department of Health World Trade Center Registry (WTCHR), lower rates of lung cancer were identified in the civilian population [19] and a significantly reduced SIR was also observed for lung cancer among civilians during the follow-up period from 2007 through 2011 (SIR 0.69, 95% CI 0.54–0.88) [42]. However, the required latency for lung cancer development may be longer than the data available in this time span. The civilian cohort at the WTC EHC is self-referred and thus not suited for the determination of the incidence rate of lung cancer. The objective of the current study was to describe lung cancer characteristics at WTC EHC.

Tobacco use, a known risk factor associated with lung cancer, was identified as a risk factor in the WTC EHC cohort. This finding is consistent with findings described in responders in which being a current smoker, although not a former smoker, was associated with lung cancer risk [43]. Overall, the chance that a man will develop lung cancer in his lifetime is about 1 in 15, for a woman, the risk is about 1 in 17 [44]. These numbers include both smokers and non-smokers. For smokers, the risk is much higher, while for non-smokers the risk is lower [44]. At the WTC EHC, many patients with lung cancer were never-smokers, including those with a diagnosis at a very young age. The median age of diagnosis for the never-smokers (62 years) was slightly lower, compared to the ever-smokers (65 years). The rates of acute WTC dust exposure defined as WTC dust cloud exposure was similar in ever- and never-smokers. Importantly, the median age of lung cancer diagnosis in the total lung cancer population was 64 years, nearly seven years younger than the median age of 71 years reported in national statistics [45]. Although these results may be due to early detection or selection bias since our population is self-referred and closely monitored, the young age at lung cancer diagnosis raises concern and suggests further investigation into underlying mechanisms causes.

Cancer occurrence differs by race/ethnicity with lower rates of lung cancer in Hispanics and Asians compared with Non-Hispanic Whites [45]. Many of lung cancer patients in the WTC EHC were Asian, particularly among the never-smokers. This finding may be due, in part, to selection bias within our population, which included many patients from areas in New York City with Asian communities.

The histologic distribution of cancers in the WTC EHC population varies from what has been described in the general population in which, about 13% of all lung cancers are small cell lung carcinoma, and 84% are non-small cell lung carcinoma [44]. In contrast, we identified fewer than 3% as small cell lung carcinoma. Approximately half of the lung cancers in the US are reported as adenocarcinoma [46,47], however, in our population, the majority (71%) of tumors were adenocarcinoma, with high rates in both never- and ever-smokers. We report squamous cell carcinomas in a higher proportion than that reported for the general population [48,49,50,51] and as expected, this histologic type was more common in the ever-smokers. Although rare in the general population, carcinoid tumors were identified in 6% of the WTC EHC population, and were especially high among WTC-exposed women (9%). Although direct comparison with the general population may be misleading due to the self-referral of our population, these histological findings are intriguing and require further confirmation in future studies with appropriate matched population samples.

Most of the lung cancers in the WTC EHC were identified at early stage. National statistics show much lower rates of lung cancer diagnosed at early stages with only 17% of lung cancers diagnosed at the local stage, 22% diagnosed with regional lymph node involvement and 57% of lung cancer cases having distant metastases [44,46]. However, most of our patients (42%) were diagnosed at local stage and only 15% had distant metastasis. We observed 64 lung cancer patients (26%) with a second primary cancer [39] which is similar to the report of second primary cancers reported in responders with thyroid cancer [18] and is higher than the data reported in the SEER database [46]. The high proportion of second primary cancers may be due to WTC exposures, genetic predisposition, increased monitoring, or a combination of these factors. The early detection and predominance of adenocarcinoma suggests that many of these patients may have been detected through screening or referral bias. However, the early stage and atypical histologic distribution warrants further evaluation for detection and survivor bias.

Importantly, our population with lung cancer included over 50% women. Women with lung cancer were slightly younger than men, with lung cancer diagnoses identified in some individuals in their 30s (youngest age of lung cancer diagnosis was 34 years for women and 38 years for men). These data are consistent with findings in the U.S. population in which lung cancer, which is rare in adolescents and young adults, is higher in women in their 30s compared to men [52]. We did not detect a difference between WTC dust cloud exposure on 11 September between women and men. Many more women were never-smokers compared to men, consistent with national cancer statistics [44]. Because of the self-selected nature of our cohort, it would not be appropriate to make direct comparisons between our population and general population. However, the suggested differences require further investigation.

There are several limitations of our study. The WTC EHC is a treatment program with self-referred patients. Thus, it does not provide information necessary to determine the cancer incidence rate. An important study limitation is our inability to distinguish cancers detected because of increased monitoring and screening of this population. Early lung cancer detection in our population may be due to a number of examinations, use of advanced diagnostic imaging, or medical examinations leading to early cancer detection. However, most of the cancers were self-referred, and thus not identified through the screening in the WTC EHC. Ideally, there is a need to supplement current databases with a cancer specimen bank in order to conduct association study to elucidate the possible connection between WTC exposures and lung as well as other cancers. There is also a need for a more objective WTC exposure assessment.

This study has important strengths. In contrast to the responders, the WTC EHC includes nearly 50% women, and is racially and ethnically diverse. This diversity is reflected in the patients with lung cancer, in whom more than 50% were women. The gender distribution allows for comparison of cancer rates and cancer behaviors between men and women, a study that is not possible in many responder populations. Our pan-cancer database enables us to identify patients who have agreed to be re-contacted, and have documentation of the location of biopsy specimens. Thus, we have the potential to re-call patients for future investigations and identify banked biospecimens, if necessary. The continued surveillance and follow-up of this population allows for improved understanding of the behavior of these cancers over time.

## 5. Conclusions

We provide an initial description of lung cancers in local community members with documented exposure to the WTC dust and fumes. This study sets the stage for future studies of lung cancer latency under environmental and other exposures. The large number of women with cancer, the younger age of lung cancer diagnosis, and the high frequency of adenocarcinomas and carcinoid tumors in women warrant further analysis, including a more detailed analysis using lung cancer biomarkers. The pathologic and histologic characteristics as well as cancer related biomarker information included in our WTC EHC Pan-Cancer Database will allow for future analyses of lung cancer characteristics, understanding the potential pathophysiological pathways of cancers and trends in the WTC-exposed survivors, and may provide additional information about association of environmental exposures and the development of lung and other cancers in this cohort.

## Figures and Tables

**Table 1:** Characteristics of lung cancer patients in the World Trade Center Environmental Health Center (WTC EHC) (*n* = 203) with smoking history in the WTC EHC.

			Smoking		
		Overall	Never (≤1 p-y)	Ever (>1 p-y)	*p*
*n*		203	76	127	
Gender, *n* (%)	Female	119 (58.6)	58 (76.3)	61 (48.0)	<0.001
	Male	84 (41.4)	18 (23.7)	66 (52.0)	
Age on 9/11 (years) (median (range))		50.7 (25.3, 74.0)	49.7 (27.5, 71.6)	51.9 (25.3, 74.0)	0.21
Age of diagnosis (years) (median (range))		64 (34, 8)	62 (38, 88)	65 (34, 89)	0.07
Race/Ethnicity, *n* (%)	White	110 (55.8)	29 (39.2)	81 (65.9)	<0.001
	Black	27 (13.7)	9 (12.2)	18 (14.6)	
	Asian	46 (23.3)	34 (45.9)	12 (9.8)	
	Hispanic	14 (7.1)	2 (2.7)	12 (9.8)	
BMI, n (%)	Normal weight (<25)	56 (38.6)	23 (50.0)	33 (33.3)	0.17
	Overweight (25–30)	51 (35.2)	13 (28.3)	38 (38.4)	
	Obese (≥30)	38 (26.2)	10 (21.7)	28 (28.3)	
Income, *n* (%)	≤$30,000/year	107 (56.0)	45 (64.3)	62 (51.2)	0.09
	>$30,000/year	84 (44.0)	25 (35.7)	59 (48.8)	
Education, *n* (%)	High school or less	78 (38.4)	32 (42.1)	46 (36.2)	0.46
	More than high school	125 (61.6)	44 (57.9)	81 (63.8)	
Dust cloud, *n* (%)	No	103 (50.7)	37 (48.7)	66 (52.0)	0.66
	Yes	100 (49.3)	39 (51.3)	61 (48.0)	
Exposure category, *n* (%)	Worker	115 (56.9)	29 (38.2)	86 (68.3)	<0.001
	Resident	74 (36.6)	44 (57.9)	30 (23.8)	
	Other	13 (6.4)	3 (3.9)	10 (7.9)	

**Table 2:** Characteristics of lung cancers in the WTC Environmental Health Center with smoking history.

			Smoking		
	Level	Overall	Never (≤1 p-y)	Ever (>1 p-y)	*p*
*n*		203	76	127	
Histology, *n* (%)	Adenocarcinoma	143 (70.4)	60 (79.0)	83 (65.4)	0.02
	Squamous Cell Carcinoma	24 (11.8)	4 (5.3)	20 (15.6)	
	Carcinoid Tumors	13 (6.4)	7 (9.2)	6 (4.7)	
	Large cell Carcinoma	3 (1.5)	1 (1.3)	2 (1.6)	
	Small Cell Carcinoma	3 (1.5)	0 (0.0)	3 (2.4)	
	Unidentified Non-Small Cell Carcinoma or Unknown	17 (8.4)	4 (5.2)	13 (10.3)	
Grade, *n* (%)	G1	32 (15.8)	14 (18.4)	18 (14.2)	0.77
	G2	44 (21.6)	16 (21.1)	28 (22.0)	
	G3	42 (20.7)	15 (19.7)	27 (21.3)	
	G4	1 (0.5)	0 (0.0)	1 (0.8)	
	GX or Unknown	84 (41.4)	31 (40.8)	53 (41.7)	
Tumor size, *n* (%)	Tis	6 (2.9)	5 (6.6)	1 (0.8)	0.21
	T1	88 (43.4)	32 (42.1)	56 (44.2)	
	T2	41 (20.2)	12 (15.7)	29 (22.8)	
	T3	14 (6.9)	4 (5.3)	10 (7.9)	
	T4	6 (2.9)	2 (2.6)	4 (3.1)	
	TX or Unknown	48 (23.7)	21 (27.7)	27 (21.2)	
Tumor Size in cm (median (range))		1.7 (0.2, 11.5)	1.8 (0.4, 5.0)	1.7 (0.2, 11.5)	0.84
Regional Lymph Node Metastasis, *n* (%)	N0	110 (54.2)	39 (51.3)	71 (55.9)	0.47
	N1	52 (25.6)	17 (22.4)	35 (27.6)	
	NX or Unknown	41 (20.2)	20 (26.3)	21 (16.5)	
Distant Metastasis, *n* (%)	M0	139 (68.5)	48 (63.2)	91 (71.6)	0.20
	M1	32 (15.7)	15 (19.7)	17 (13.4)	
	MX or Unknown	32 (15.8)	13 (17.1)	19 (15.0)	
Stage, *n* (%)	0	6 (3.0)	5 (6.6)	1 (0.8)	0.08
	I	86 (42.3)	29 (38.2)	57 (44.8)	
	II	21 (10.3)	7 (9.2)	14 (11.0)	
	III	26 (12.8)	7 (9.2)	19 (15.0)	
	IV	32 (15.8)	15 (19.7)	17 (13.4)	
	Unknown	32 (15.8)	13 (17.1)	19 (15.0)	
Anatomic location, *n* (%)	Right upper lobe	50 (24.6)	18 (23.7)	32 (25.2)	0.60
	Right middle lobe	8 (3.9)	3 (3.9)	5 (3.9)	
	Right Lower Lobe	17 (8.4)	10 (13.2)	7 (5.5)	
	Right unspecified	35 (17.2)	14 (18.4)	21 (16.5)	
	Left upper lobe	32 (15.8)	9 (11.8)	23 (18.1)	
	Left Lower Lobe	18 (8.9)	6 (7.9)	12 (9.5)	
	Left unspecified	39 (19.2)	15 (19.8)	24 (18.9)	
	Bilateral	1 (0.5)	0 (0.0)	1 (0.8)	
	Unknown	3 (1.5)	1 (1.3)	2 (1.6)	

**Table 3:** Characteristics of lung cancer patients (*n* = 248) classified by gender in the WTC EHC.

		Sex		
	Level	Female	Male	*p*
***n***	248	142	106	
Age on 9/11 (median (range))		49.2 (27.5, 69.4)	52.7 (25.3, 74.0)	0.002
Age of diagnosis, years (median (range))		62 (34, 85)	66 (38, 89)	0.002
Race/Ethnicity, *n* (%)	Hispanic	5 (4.2)	10 (12.3)	0.03
	White	63 (52.9)	48 (59.3)	
	Black	22 (18.5)	6 (7.4)	
	Asian	29 (24.4)	17 (21.0)	
BMI, *n* (%)	Normal weight (<25)	40 (46.0)	18 (29.5)	0.09
	Overweight (25–30)	25 (28.7)	27 (44.3)	
	Obese (≥30)	22 (25.3)	16 (26.2)	
Income, *n* (%)	≤$30,000/year	69 (60.0)	41 (51.9)	0.30
	>$30,000/year	46 (40.0)	38 (48.1)	
Education, *n* (%)	High school or less	42 (34.4)	36 (42.9)	0.24
	More than high school	80 (65.6)	48 (57.1)	
Dust cloud exposure, *n* (%)	No	64 (52.5)	41 (48.8)	0.67
	Yes	58 (47.5)	43 (51.2)	
Exposure category, *n* (%)	Resident	51 (42.1)	24 (28.6)	0.12
	Student	4 (3.3)	1 (1.2)	
	Worker	61 (50.4)	56 (66.7)	
	Other	5 (4.1)	3 (3.6)	
Pack-year, *n* (%)	≤5 pack-year	64 (53.8)	22 (26.2)	<0.001
	>5 packyear	55 (46.2)	62 (73.8)	
Smoking History, *n* (%)	Never (≤1 p-y)	58 (40.8)	18 (17.0)	<0.001
	Former smoker (>1 p-y, stopped)	53 (37.3)	60 (56.6)	
	Current smoker (>1 p-y, continue)	8 (5.6)	6 (5.7)	

**Table 4:** Characteristics of lung cancers with gender classification in the WTC Environmental Health Center.

			Sex		
	Level	Overall	Female	Male	*p*
*n*		248	142	106	
Histology, *n* (%)	Adenocarcinoma	176 (71.0)	107 (75.3)	69 (65.1)	0.001
	Squamous cell carcinoma	26 (10.5)	8 (5.6)	18 (17.0)	
	Carcinoid tumors	16 (6.5)	13 (9.1)	3 (2.8)	
	Small cell carcinoma	6 (2.4)	2 (1.4)	4 (3.8)	
	Large cell carcinoma	3 (1.2)	1 (0.7)	2 (1.9)	
	Unidentified subtype of Non-small cell carcinoma or unknown	21 (8.4)	11 (7.7)	10 (9.4)	
Grade, *n* (%)	G1	43 (17.3)	30 (21.1)	13 (12.3)	0.23
	G2	53 (21.4)	30 (21.1)	23 (21.7)	
	G3	51 (20.6)	27 (19.0)	24 (22.6)	
	G4	1 (0.4)	0 (0.0)	1 (0.9)	
	GX or Unknown	100 (40.3)	55 (38.8)	45 (42.5)	
Tumor size, TNM, *n* (%)	Tis	8 (3.2)	8 (5.6)	0 (0.0)	0.006
	T1	105 (42.3)	66 (46.5)	39 (36.8)	
	T2	53 (21.4)	21 (14.8)	32 (30.2)	
	T3	16 (6.5)	7 (5.0)	9 (8.5)	
	T4	8 (3.2)	4 (2.8)	4 (3.8)	
	TX or Unknown	58 (23.4)	36 (25.3)	22 (20.7)	
Tumor size, cm median (range)		1.8 (0.2, 11.5)	1.7 (0.4, 6.5)	2.0 (0.2, 11.5)	0.03
Regional lymph node metastasis, *n* (%)	N0.	136 (54.8)	76 (53.5)	60 (56.6)	0.81
	N1	63 (25.4)	37 (26.1)	26 (24.5)	
	NX or Unknown	49 (19.8)	29 (20.4)	20 (18.9)	
Distant Metastasis, *n* (%)	M0	170 (68.6)	94 (66.2)	76 (71.7)	0.16
	M1	38 (15.3)	25 (17.6)	13 (12.3)	
	MX or Unknown	40 (16.1)	23 (16.2)	17 (16.0)	
Stage, *n* (%)	0	8 (3.3)	8 (5.6)	0 (0.0)	0.06
	I	105 (42.3)	55 (38.7)	50 (47.1)	
	II	27 (10.9)	14 (9.9)	13 (12.3)	
	III	30 (12.1)	17 (12.0)	13 (12.3)	
	IV	38 (15.3)	25 (17.6)	13 (12.3)	
	Unknown	40 (16.1)	23 (16.2)	17 (16.0)	
Anatomic Location, *n* (%)	Right upper lobe	62 (25.0)	32 (22.5)	30 (28.4)	0.01
	Right middle lobe	10 (4.0)	8 (5.6)	2 (1.9)	
	Right lower lobe	20 (8.1)	12 (8.5)	8 (7.5)	
	Right unspecified	43 (17.4)	26 (18.3)	17 (16.0)	
	Left upper lobe	38 (15.3)	21 (14.8)	17 (16.0)	
	Left Lower Lobe	26 (10.5)	9 (6.4)	17 (16.0)	
	Left unspecified	43 (17.3)	30 (21.1)	13 (12.3)	
	Bilateral	2 (0.8)	0 (0.0)	2 (1.9)	
	Unknown	4 (1.6)	4 (2.8)	0 (0.0)	

## Data Availability

The datasets are not publicly available, but de-identified and anonymized information is potentially available upon reasonable request.

## Supplementary Materials

The following are available online at https://www.mdpi.com/1660-4601/18/5/2689/s1. Table S1. Number of multiple primary cancers with lung cancer in WTC EHC as of 31 December 2019; Table S2. Characteristics of second diagnosed primary lung cancers in WTC; Figure S1. Distribution of age of lung cancer diagnosis in never and ever smokers separated by sex.
