# Supplementary material for: Lung Cancer Characteristics in the World Trade Center Environmental Health Center
Source: Int J Environ Res Public Health. 2021 Mar 7;18(5):2689. doi: 10.3390/ijerph18052689 (PMC7967411; doi:10.3390/ijerph18052689)
Supplement: Supplementary file 1 [file ijerph-18-02689-s001.pdf]

Table S1. Number of multiple primary cancers with lung cancer in WTC EHC as of 31 December 2019.

Cancer	*n*	%
Lung	15	23
Thyroid	12	19
Prostate	10	16
Breast	8	12
Colon rectum	5	8
Lymphoma	4	6
Melanoma	2	3
Urinary	2	3
Brain and spinal cord	1	1.5
Myeloproliferative	1	1.5
Myeloma	1	1.5
Myelodysplastic	1	1.5
Neuroendocrine	1	1.5
Pancreas	1	1.5
Soft tissue	1	1.5
Ovary	1	1.5
Corpus uteri	1	1.5
Liver	1	1.5
Extrahepatic bile	1	1.5
Kidney	1	1.5
Head	1	1.5
Small intestine	1	1.5

Table S2. Characteristics of second diagnosed primary lung cancers in WTC EHC.

	Level	Overall
n		15
**Laterality, *n* (%)**	Left	2 (13.3)
	Right	10 (66.7)
	Unknown	3 (20.0)
**Grade, *n* (%)**	G1	1 (6.6)
	G2	4 (26.7)
	G3	0 (0.0)
	G4	0 (0.0)
	GX or Unknown	10 (66.7)
**Tumor size, *n* (%)**	T1	10 (66.7)
	T2	0 (0.00)
	T3	2 (13.3)
	T4	0 (0.0)
	TX or Unknown	3 (20.0)
Tumor Size in cm (median [range])		1.25 [0.4, 10]
Regional Lymph Node Metastasis, *n* (%)	N0	10 (66.7)
	N1	3 (20.0)
	NX or Unknown	2 (13.3)
Distant Metastasis, *n* (%)	M0	12 (80.0)
	M1	1 (6.7)
	MX or Unknown	2 (13.3)
Stage (%)	I	9 (60.0)
	II	2 (13.3)
	III	1 (6.7)
	IV	1 (6.7)
	Unknown	2 (13.3)

Histology (%)	Adenocarcinoma	9 (60.0)
	Squamous Cell Carcinoma	3 (20.0)
	Carcinoid Tumors	1 (6.7)
	Large cell Carcinoma	1 (6.7)
	Small Cell Carcinoma	1 (6.7)

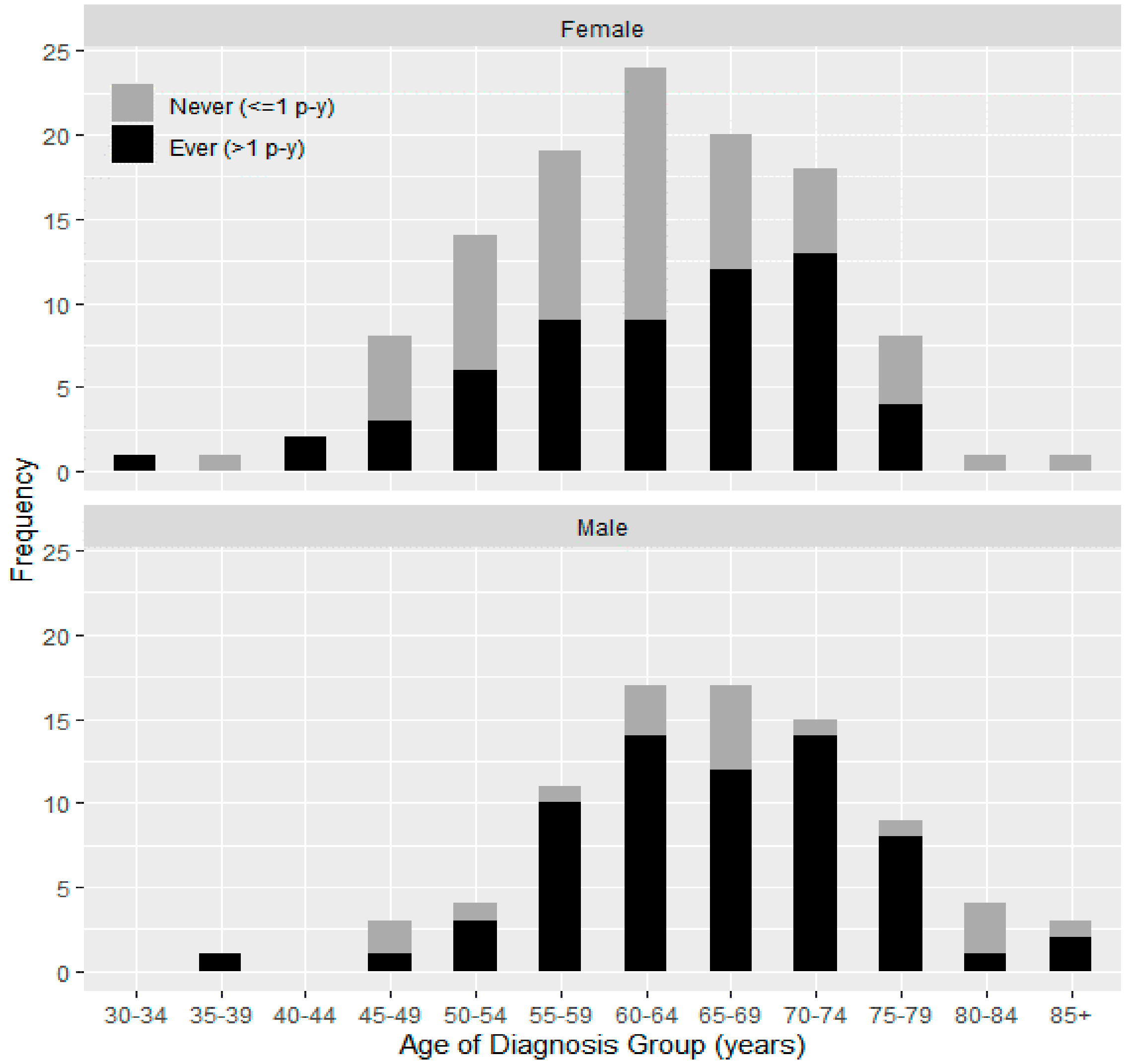

Figure S1. Distribution of age of lung cancer diagnosis in never and ever smokers separated by sex.
